# Single-dose oral administration of drug-loaded magnetic 3D-printed microbullets for eradication of *Helicobacter pylori*

**DOI:** 10.1016/j.ajps.2024.101013

**Published:** 2025-01-03

**Authors:** Hua Xie, Dongdong Liu, Jintao Shen, Wenrui Yan, Meng Wei, Yingbao Sun, Yubao Fang, Bochuan Yuan, Pei Deng, Yiguang Jin

**Affiliations:** a Department of Pharmaceutical Sciences, Beijing Institute of Radiation Medicine, Beijing 100850, China; b Guangdong Pharmaceutical University, Guangzhou 510006, China; c China Rehabilitation Science Institute, China Rehabilitation Research Center, Key Laboratory of Neural Injury and Rehabilitation, Beijing 100068, China; d Department of Gastroenterology, Second Clinical Medical College of Beijing University of Chinese Medicine (Dongfang Hospital), Beijing 100078, China

**Keywords:** 3D printing, *Helicobacter pylori*, Magnetic guidance, Clarithromycin, Drug delivery system, Microbullet

## Abstract

Infections of *Helicobacter pylori* (*H. pylori*) affect 42.1 % of the Chinese population and 43.1 % of the world population. *H. pylori* inhabits the mucous sublayer at the pylorus, leading to gastric ulcers, gastritis, and even cancer. Oral antibiotics are usually used to treat *H. pylori* infections, whereas traditional quadruple therapy has side effects including headaches, nausea, diarrhea, intestinal dysbacteriosis, antibiotic resistance, and repeat infections. Here, a drug-loaded magnetic microbullet was designed to realize long-term retention in the stomach for one-shot treatment for *H. pylori* infections. It comprises a hollow cylinder wherein eight microneedles homogenously distribute at the top and several round pores located at the bottom. It was three-dimensional (3D)-printed by stereolithography. A clarithromycin (CAM) ground mixture (CGM) was prepared to improve solubility. Enough CGM powders were filled into the cylinder, covered by a small round magnet, and sealed to form a CAM-loaded magnetic microbullet (CMMB). CAM continually released from CMMBs for >24 h. With outside magnetic guidance, an oral CMMB targeted the pylorus site and the microneedles immediately headed into the mucosa followed by long-term local drug release. The *in vitro* and *in vivo* safety of CMMBs was confirmed, where their swelling rates were low, and the oral CMMB was finally completely evacuated. An oral CMMB was administered to *H. pylori*-infected mice and maintained in the stomach for 36 h with magnetic guidance, and the successful eradication of *H. pylori* was confirmed after single-dose administration. Oral CMMBs are a convenient medication for the eradication of *H. pylori*.

## Graphical abstract

**Figure:**
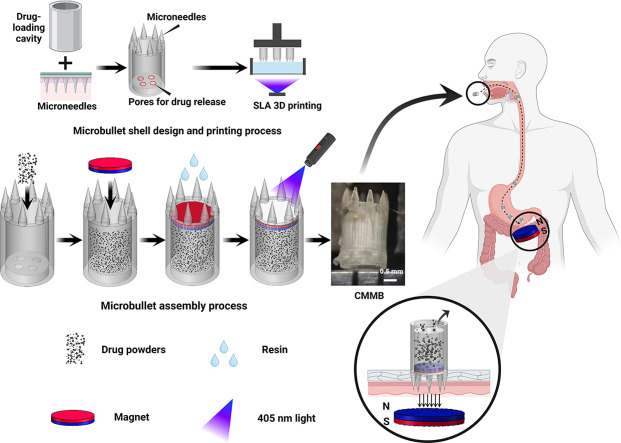

## 1 Introduction

*Helicobacter pylori* (*H. pylori*), a gram-negative microaerobic bacterium inhabiting the mucous sublayer at the pylorus, is the major cause of many stomach diseases, such as gastric ulcers, gastritis, and even stomach cancer, due to the expression of virulence-associated proteins cytotoxin-associated gene A (CagA) and vacuolating cytotoxin A (VacA) [[1], [2], [3]]. The global prevalence of *H. pylori* infections was 43 % between 2011 and 2022, placing a significant clinical and public health burden [4]. Oral medications have difficulty maintaining an effective drug concentration in the stomach due to the short gastric retention time (GRT) and the absence of drug targeting and stability [5,6]. The current clinical treatment of *H. pylori* infections aims to inhibit *H. pylori* using multi-dose quadruple therapy, including bismuth salts, proton pump inhibitors, and two antibiotics [7,8]. However, the traditional multi-dose quadruple therapies present challenges, despite their effectiveness, such as headaches, nausea, diarrhea, intestinal dysbacteriosis, and other side effects from overuse of antibiotics, which may lead to poor patient compliance and increased antibiotic resistance [[9], [10], [11]]. In contrast, single-dose eradication therapies have advantages in improving patient compliance and reducing side effects, which decreases the emergence of drug resistance. However, single-dose therapies require a higher dose of antibiotics to ensure efficacy, extend the GRT of the drug, maintain the concentration of the drug in the stomach, and improve the clearance of *H. pylori*. Therefore, a safe and highly effective drug delivery system that can eradicate *H. pylori* with a single dose is urgently needed.

In many treatment needs, prolonging GRT and reducing the frequency of administration are the best schemes to improve the clearance rate of *H. pylori* [12]. The gastric retentive drug delivery system (GRDDS), as a drug delivery device, has been used for site-specific drug release in a controlled manner to ensure local or systemic action in the gastrointestinal tract [13]. The commonly used GRDDSs include floating, expandable, bio-adhesive, and magnetic systems, significantly extending drug release duration, increasing drug bioavailability, and improving patient compliance and pharmacotherapy effectiveness [[14], [15], [16], [17]]. Compared to other types of GRDDSs, magnetic GRDDSs with positioning capabilities are more suitable for the eradication of *H. pylori*, because the colonizing sites of *H. pylori* are in the mucous sublayer. However, the residence time, targeting location, and drug-loading efficiency of traditional magnetic GRDDSs are limited due to the lack of structural design [[18], [19], [20]]. Therefore, the development of a magnetic GRDDS with a special structure to enhance drug loading efficiency and GRT is critically necessary.

Microneedles are widely used for drug delivery both *in vivo* and *in vitro* because of their superior penetration ability and painless injection characteristics [[21], [22], [23]]. However, the traditional method of incorporating drugs into the microneedle matrix has limited drug loading capacity. Although hollow microneedles present an improvement in drug loading efficiency, they are prone to being blocked by tissues, which decreases drug delivery efficiency. Consequently, we delegate the drug-loading function to an additional cavity structure. The microneedles are exclusively designed for piercing the gastric mucosa, thereby enhancing the ability of the drug delivery system to resist gastric emptying, achieving high drug loading, and prolonging the GRT. The combination of microneedles with a cavity could achieve high drug loading while extending GRT. However, the complex structure made it difficult to prepare for traditional manufacturing technologies. Three-dimensional (3D) printing technologies are renowned for their capability to fabricate intricate structural devices [[24], [25], [26]]. They are outstanding in the production of small and complex drug delivery devices due to their advantages of precise control, rapid curing, and high resolution [[27], [28], [29]]. Therefore, we printed a magnetic GRDDS with stereolithography (SLA) 3D printing and aimed to overcome the current challenges of high drug loading and frequent administration required for the treatment of *H. pylori* infection.

**Fig. 1:**
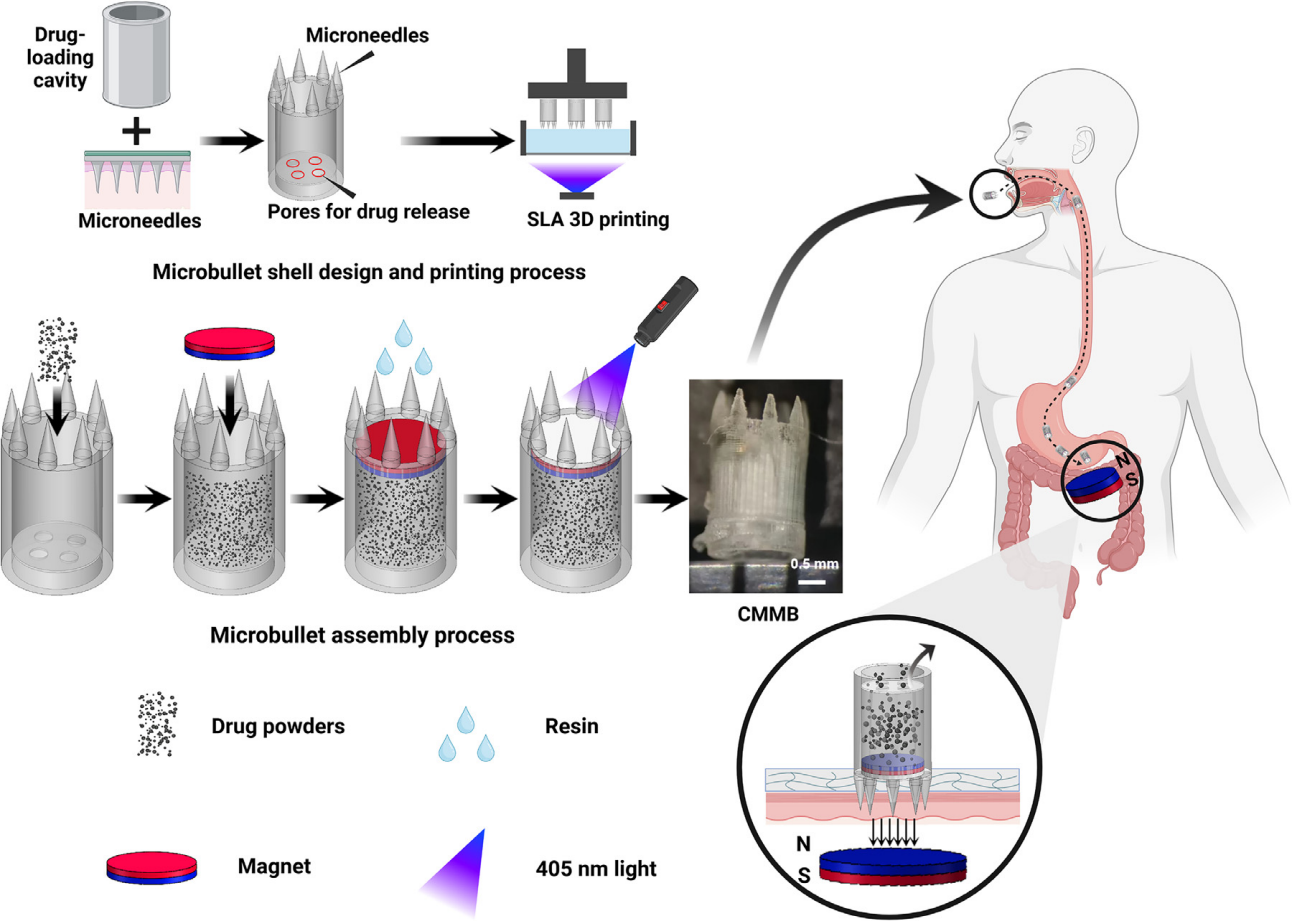
Schematic illustrations of design, printing, assembly process, and drug release of CMMBs.

Here, a clarithromycin-loaded magnetic microbullet (CMMB) was prepared and orally administered to eradicate *H. pylori* (Fig. 1). The structural design, drug release, mechanical properties, and biocompatibility of CMMBs were thoroughly evaluated. CMMBs not only anchored the microbullet tips to the pylorus under the external magnetic force but also continually released clarithromycin (CAM) so that long-term local high drug concentrations ensured the eradication of *H. pylori*. Once the outside magnet is withdrawn, CMMBs can be completely evacuated from the body.

## 2 Materials and methods

### 2.1 Materials

CAM was purchased from Shanghai Bide Pharmaceutical Technology Co., Ltd. (Shanghai, China). A CAM ground mixture (CGM) was prepared with CAM and 2-O-α-d-glucopyranosyl-l-ascorbic acid (AA-2G) for enhancing solubility. The preparation and characterization details of CGM were described in the supplementary materials. Dental surgical guide (SG) resin was purchased from Formlabs Co., Ltd. (Millbury, USA). Roswell Park Memorial Institute (RPMI) 1640 media were obtained from Gibco Life Technologies (Carlsbad, USA). Streptomycin sulfate and trypsin-EDTA were provided by Beijing Solarbio Science & Technology Co., Ltd. (Beijing, China). Fetal bovine serum (FBS) was purchased from Zhejiang Tianhang Biotechnology Co., Ltd. (Huzhou, China). Cell Counting Kit-8 (CCK-8) was provided by Dojindo Beijing Co., Ltd. (Beijing, China). *H. pylori* Sydney strain 1 (SS1) was provided by the Department of Gastroenterology, Peking University First Hospital. Brain and heart infusion media (BHI) were purchased from Qingdao Hope Bio-Technology Co., Ltd. (Qingdao, China). Ciprofloxacin, rhodamine B, and tetracycline hydrochloride were provided by Beijing InnoChem Science & Technology Co., Ltd. (Beijing, China). Universal blue SYBR qPCR master mix (2×) was purchased from Wuhan Servicebio Technology Co., Ltd. (Wuhan, China).

A normal murine fibroblast L929 cell line was obtained from the Cell Bank of the Chinese Academy of Sciences (Shanghai, China) and cultured in the RPMI 1640 supplemented with 10 % FBS at 37 °C in a humidified 5 % CO_2_ atmosphere. Male C57BL/6J mice (body weight, 20–22 g) were provided by the Beijing Vital River Laboratory Animal Technology Co., Ltd. (Beijing, China). The mice were housed in a specific pathogen-free environment with a regular light/dark cycle and allowed unrestricted access to food and water. All experiments were performed according to the Ethical Committee of the Beijing Institute of Radiation Medicine for the Care and Use of Laboratory Animals.

### 2.2 Design of microbullets

**Fig. 2:**
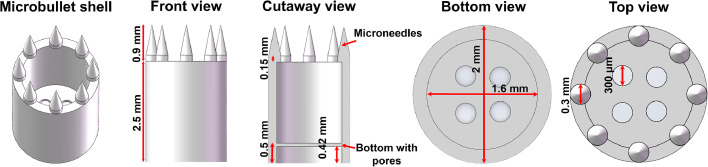
Illustration of the microbullet shell (Type 300/4) model with the front, cutaway, bottom, and top views.

A CMMB consisted of a microbullet shell, an NdFeB magnet (1350 GS, a diameter of 1 mm, and a height of 0.5 mm, Lala Magnetic Material Development Co., Ltd., Shenzhen, China), and loaded drugs. The main body of microbullet shells was a hollow cylinder with several round pores on the bottom. The microbullet tip consisted of eight homogenously distributing microneedles (0.9 mm high, 0.3 mm in diameter). The hollow cylinder had a height of 2.5 mm, an external diameter of 2 mm, and an internal diameter of 1.6 mm (Fig. 2). Four types of microbullets were designed with four pore combinations: the diameter of 200 µm and 2 pores or 4 pores, named Type 200/2 and Type 200/4, respectively, or the diameter of 300 µm and 2 pores or 4 pores, named Type 300/2 and Type 300/4, respectively.

### 2.3 3D printing of microbullet shells

All the microbullet shell models were established with the Solidworks 2021 software (Dassault Systemes, Pairs, USA). The model files were saved with the STL style and exported to the slicer files with the CWS format after being processed by the NovaMaker software (Shenzhen Nova Intelligent Technology Co., Ltd., Shenzhen, China). Microbullet shells were printed with the Dental SG resin using the Nova 3D printer (Bene4, Shenzhen Nova Intelligent Technology Co., Ltd., Shenzhen, China) armed with a 405 nm laser. The 3D printing parameters were as follows: the layer thickness of 40 µm, the base plate layers of 10, the base plate exposure time of 40 s, and the subsequent layer exposure time of 9 s. The resin residues on the surface of microbullet shells were washed with absolute ethanol. Microbullet shells were dried at 60 °C for 1 h in an electric blast drying oven (BGZ-140, Shanghai Boxun Industrial Co., Ltd., Shanghai, China) and cured for 1 h under a 405-nm lamp (EFL-LS-1600–405, Suzhou Yongqinquan Intelligent Equipment Co., Ltd., Suzhou, China) at room temperature.

### 2.4 Scanning electron microscopy

A microbullet shell was adhered to the object stage with conductive tape. Then, a layer of gold was sputtered on the surface of microbullet shells with a gold spray instrument (SBC-12, Beijing Zhongke Technology Co., Ltd., Beijing, China). The morphology of microbullet shells was observed under a scanning electron microscope (SEM, EmCrafts CUBE II, Gyeonggi-do, Korea).

### 2.5 Investigation of the mechanical strength of microbullet tips

A microbullet shell was put vertically on the metal table of a universal mechanical testing machine (INSTRON 5982, Instron Engineering Corporation, Massachusetts, USA), with the microbullet tips facing up. Compression was tested at a speed of 0.15 mm/min. The test was stopped when the displacement reached 800 µm. Furthermore, the displacement and force curves of microbullet tips were recorded.

### 2.6 Swelling test

The pH of the microenvironment around the *H. pylori* colonization position is raised to 5–6 due to the *H. pylori*-secreting special urease that can convert urea into ammonia [30]. The simulated gastric fluid and intestinal fluid pH values are 1.2 and 6.8, respectively [31]. Therefore, the swelling behaviors of microbullet shells were evaluated at pH 1.2, 5.0 and 6.8. We prepared cylinders (4 mm × 4 mm) with the same materials as the microbullets and the method described in Section 2.4. The cylinders were used to investigate the swelling rate of microbullet shell materials. All the cylinders were weighed as the initial weight and incubated at 37 °C in the pH 1.2 solutions for 12 h, followed by transfer to the pH 5.0 solutions for 36 h, and finally immersed in the pH 6.8 solutions for 12 h, respectively. The cylinders were taken out at the predetermined time of 4, 8, 12, 16, 20, 24, 36, 48, 52, 56 and 60 h, respectively, followed by wiping with filter paper. The dried cylinders were weighed as the wet weight. The swelling rates were calculated as Eq. (1):

(1) $$Swelling\;rate\;\left(\%\right)=\frac{M_{1}-M_{0}}{M_{0}}\times 100\%$$

where *M_0_* and *M_1_* indicated the initial weight and wet weight of the cylinders, respectively.

### 2.7 *In vitro* antibacterial experiment

The antibacterial experiment was used to investigate the ability of CGM to resist *H. pylori*. CAM and CGM were dissolved in dimethyl sulfoxide and the BHI medium (containing 10 % FBS), respectively. The solutions were diluted with the BHI medium to achieve CAM concentrations of 0.065, 0.125, 0.25 and 0.5 µg/ml, respectively. The dilution (990 µl) was mixed with an *H. pylori* suspension (10^9^ CFU/ml, 10 µl), followed by incubation at 37 °C with 5 % O_2_ for 24 h. The solution was 100-fold diluted with the BHI medium, followed by being transferred to blood agar plates. The number of colonies in the plates was recorded after incubation at 37 °C and 5 % O_2_ for 48 h. The minimal inhibitory concentration (MIC) value was the minimum drug concentration in the sterile growth plate.

### 2.8 Loading drugs and assembly of the microbullets

The cavity of microbullet shells was filled with CGM powders, and then a 1350 GS polarizing magnet was placed horizontally to cover the powders and sealed with 0.25 µl Dental SG resin under a 405-nm lamp. The assembly process was shown in Fig. S3. The *in vitro* CAM continual release from CGM-loaded microbullets was confirmed (Supplementary Materials 1.2). Compared to CAM-loaded microbullets, CGM-loaded microbullets released drugs faster and more stable. Moreover, the Type 300/4, *i.e.*, the pore combination with 4 pores and 300 µm in diameter, had the highest release rates among all the microbullets (Fig. S4). Therefore, the Type 300/4 was chosen as the optimal CMMB for the next research.

### 2.9 Investigation of the GRT of microbullets

The *in vitro* magnetic guiding effect of microbullets was investigated. A magnet was placed under an agar gel brick. A tweezer clamping a microbullet was hung above the brick. When the tweezer was unclamped, the movement of the microbullet was observed. After the microbullet was removed, the trace on the brick was observed. The whole process was videoed.

Twenty-four C57BL/6J mice were randomly divided into two groups: the magnetic guide (MG) and the non-magnetic guide (NMG) groups. A microbullet was smeared with glycerin, placed vertically in the throat of the mouse, and gently pushed into the stomach with a gavage needle. Above the abdomen of the mice in the MG group, a cylindrical permanent magnet (3800 GS with a diameter of 5 mm and a height of 3 mm, Lala Magnetic Material Development Co., Ltd., Shenzhen, China) was located at the external position of the stomach to control the movement of the microbullet to the pylorus. A microbullet was administered to the mice in the NMG group without an external magnet. The position of the microbullets in the gastrointestinal tract was observed under a portable X-ray machine (ATF/X-50/75, Weihai Aitif Medical Equipment Manufacturing Co., Ltd., Weihai, China) at the predetermined time points (8, 12, 16, 20 and 24 h). The mice were anesthetized with isoflurane for administration and observation under X-rays. Iohexol solutions (0.3 ml, 20 %) were intragastrically (i.g.) administered to the mice to facilitate observation of the stomach. The mice were sacrificed, and the gastrointestinal tissues were excised and imaged. Moreover, in the *in vivo* experiment in Section 2.13, a tweezer removed the microbullet from the stomach and then unclamped it. The behavior of the microbullet was observed under external magnetic guidance. The whole process was videoed.

### 2.10 Investigation of drug *in vivo* distribution behavior

Rhodamine B (RhB), as a fluorescent probe, was used for imaging to simulate the drug distribution behavior *in vivo*. A microbullet was filled with RhB powders (2 mg) and assembled with a magnet (a diameter of 1 mm and a height of 0.5 mm, 1350 GS) to form a RhB-loaded magnetic microbullet (RMMB). RMMBs were i.g. administered to the mice as described in Section 2.10 after fasting for 12 h. The gastrointestinal tissues of the mice in the MG and NMG groups were excised and imaged under an *in vivo* imaging system (ABL-X5, Tanon Science & Technology Co., Ltd., Shanghai, China) at the predetermined time points of 4, 8, 12 and 24 h.

### 2.11 Evaluation of the microbullet safety *in vitro*

Cylinders were prepared as described in Section 2.7 and used to investigate the biocompatibility of microbullet shells by the EN ISO 10993–12:2021 guideline (when the thickness exceeds 1 mm, the extraction ratio is defined as surface area to volume, equaling 3 cm²/ml). Eighteen cylinders were immersed in the RPMI 1640 medium (4.5 ml) supplemented with 10 % FBS and 1 % penicillin/streptomycin, and then shaken at 37 °C and 100 rpm in a thermostatic oscillator for 72 h to obtain cylinder immersing solutions. The immersing solution was 1–8-fold diluted with the same medium. L929 cells were used to evaluate the toxicity of the diluted solutions. L929 cells were seeded in 96-well plates (5,000 cells/well) and incubated at 37 °C for 12 h. Then the cells were co-incubated with the diluted solutions for 24 h. After the wells were washed twice with phosphate-buffered solutions (PBS, pH 7.4), the viability of L929 cells was measured with a cell counting kit (CCK-8), and the absorbance at 450 nm was detected using the microplate reader 1 h post incubation. L929 cell viability was calculated as Eq. (2):

(2) $$Cell\;viability\;\left(\%\right)=\frac{A_{sample}-A_{blank}}{A_{control}-A_{blank}}\times 100\%$$

### 2.12 Evaluation of the microbullet safety *in vivo*

A microbullet was i.g. administered into the stomach according to the method described in Section 2.10. An external magnet was fixed where the microbullet reached for 10 min and then removed. The gastric tissues were excised and evaluated with hematoxylin and eosin (H&E) staining after the microbullets were administered for 10 min and 7 d, respectively. Similarly, the Type 300/4 CGM-loaded microbullet (approximately 0.9 mg CAM per microbullet) was retained in the stomach for 36 h before the external magnet was removed. The heart, liver, spleen, lungs, kidneys, and stomach tissues were excised and evaluated with H&E staining after 7-d administration to further investigate the *in-vivo* biocompatibility of the microbullets.

### 2.13 Establishment of *H. pylori*-infected mouse models

C57BL/6J mice were randomly divided into the healthy and model groups. The mice in the model group, followed by post-fasting for 12 h, received an antibiotic mixture (0.3 ml) including 1 % ciprofloxacin, 1 % tetracycline hydrochloride, and 0.5 % streptomycin sulfate, which was i.g. administered once daily for 5 d. On Day 6, a NaHCO_3_ solution (2 %, 0.3 ml) was i.g. administered to the mice after fasting for 12 h. After 1 h, a fresh *H. pylori* suspension (10^9^ CFU/ml, 0.3 ml) was i.g. administered to the mice once daily for 30 d. For the mice in the healthy group, saline of equal volume was i.g. administered. Two weeks after the last administration, three mice were randomly selected in every group, followed by fasting for 12 h and sacrificed. The whole stomach was excised and cut into two sections from the lesser gastric curvature to the greater gastric curvature. Half of the stomach tissue was used for histopathological examination. Another stomach section was used for the quantitative study of the specific bacterial genes of *H. pylori*.

### 2.14 Pharmacodynamic study

The pharmacodynamic study had 5 stages and continued for 103 d (Fig. 5A). Stage I was to remove the protobacteria from Day 0 to 5. Stage II was to establish *H. pylori-*infected mouse models from Day 6 to 35. Stage III was to verify the model on Day 49. The details of the above three stages are described in Section 2.14. After 2 months of *H. pylori* inhabiting [32], Stage IV was initiated on Day 95 with the administration, and the permanent magnet was fixed on the outside of the stomach in the CMMB group until 36 h post-administration. In Stage V, *i.e.*, the last stage, the therapeutic efficiency of CMMB to eradicate *in vivo H. pylori* was thoroughly investigated on Day 103.

The *H. pylori*-infected mice were randomly divided into the model, CAM, CGM, and CMMB groups (*n* = 5). CAM suspensions (0.3 ml per mouse, 3 mg/ml) were i.g. administered to the mice in the CAM group. CGM solutions (0.3 ml per mouse, eq. to 3 mg/ml CAM) were i.g. administered to the mice in the CGM group. The mice in the model group were i.g. administered with normal saline (0.3 ml per mouse). The mice in the CMMB group were anesthetized with isoflurane before the CMMB administration (approximately 0.9 mg CAM per CMMB). The external magnet was removed after 36 h post CMMB administration, and the above operations in the CMMB group were performed under the X-ray machine. All the mice were fasted for 6 h before administration and only took egg whites within 36 h post-administration. During the experimental period, the living status and body weight of mice were carefully observed and recorded. The stomach was cut into two sections, one of which was used for DNA extraction. The effect of *H. pylori* clearance was assessed by a quantitative real-time polymerase chain reaction (qPCR). The other section was used for histopathological examination.

### 2.15 qPCR determination of *H. pylori*

The linear equation between the logarithmic values of *H. pylori* concentrations and the Ct values were determined and calculated using *H. pylori* gene-specific primers (F: 5’- GCGACCTGCTGGAACATTAC-3’; R: 5’-CGTTAGCTGCATTACTGGAGA-3’) [33]. The *H. pylori* gene-specific primers were used to quantify the *H. pylori* in the stomach. The quantification of the gastric DNA extractions as a template was performed using the following qPCR reaction system: the qPCR mixture contained 10 µl Universal Blue SYBR qPCR Master Mix (2×), 0.4 µl forward primer (10 µM), 0.4 µl reverse primer (10 µM), 1 µl template and 8.2 µl sterile and enzyme-free water in the final volume of 20 µl. The experimental parameters for qPCR were as follows: 95 °C for 30 s (a cycle); 95 °C for 15 s, 60 °C for 30 s (40 cycles); 95 °C for 10 s, 75 °C for 60 s, and 95 °C for 60 s (a cycle). All the experiments were performed in triplicate.

### 2.16 Histopathological examination

The stomach tissues were immersed in 4 % paraformaldehyde for 24 h and then embedded in paraffin. Then, the stomach tissues were cut in the longitudinal direction and separately stained with H&E and Warthin-starry (W-S). W-S staining is a specific staining method to identify *H. pylori*. The pathological sections were observed under a microscope (CX33, Olympus Corp., Tokyo, Japan) for the evaluation of stomach tissue injuries and *H. pylori* infection status.

### 2.17 Statistical analysis

All data were expressed as mean ± standard deviation (SD). The results were calculated statistically using the SPSS 19.0 software (IBM, Chicago, USA). One-way analysis of variance (ANOVA) with the LSD test was used to identify differences between the data.

## 3 Results and discussion

### 3.1 Design and characteristics of 3D-printed microbullet shells

The CMMB comprises a microbullet shell, a magnetic base, and loaded drugs, where the microbullet shell plays a key role [34]. We predicted that the microbullet tip would likely insert into the mucosal layer of the stomach if it adopted a proper direction under an external force.

**Fig. 3:**
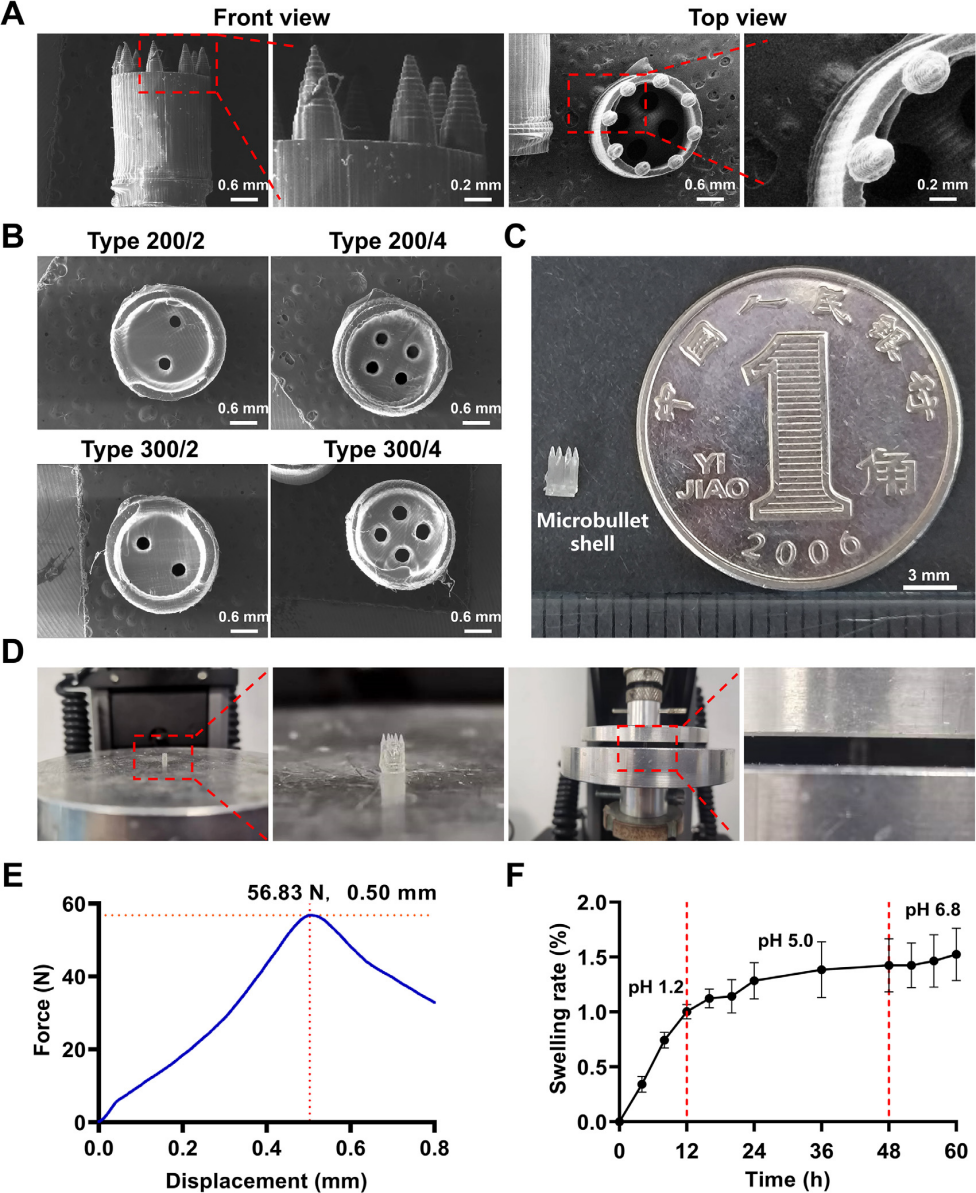
Characteristics of 3D-printed microbullet shells. The SEM images of the microbullet shells with the front and top views (A) and the bottom views (B); (C) The appearance of a microbullet shell and a coin. (D) The microbullet shell is vertically fixed in an electronic universal testing machine for the mechanical test. (E) The force-displacement curve of the microneedles on the microbullet shell (Type 300/4). (F) The swelling rates of the 3D-printed cylinders at pH 1.2, 5.0 and 6.8.

The complicated structure of microbullets makes them very difficult to produce using traditional manufacturing processes. However, 3D printing technologies provide an opportunity to produce the microbullet. Compared to other printing methods, SLA 3D printing is more suitable for the printing of microbullets because precise structures can be manufactured. Therefore, SLA 3D printing was chosen to fabricate the microbullet shell in a single step. Eight microneedles of the same size (700 µm high, 300 µm in diameter) were evenly distributed at the top circle of the cylinder (Fig. 3A) to ensure their insertion into the mucosal layer, which helps to prolong the GRT of the microbullets. The hollowed cylinder's cavity was large enough to increase the drug loading. This not only decreases the frequency of dosing but also enables the replacement of the drug at any time to align with the treatment regimen, preventing the development of resistance. An important design was the pores at the microbullet bottom, which were used for controlling the release rate of loaded drugs. Four types of pore combinations were prepared, including Type 200/2, Type 200/4, Type 300/2 and Type 300/4, which meant different pore sizes and numbers (Fig. 3B). We aim to achieve long-term sustained drug release to meet the needs of *H. pylori* treatment by adjusting the pore area and number to regulate the drug release rate. To get the relationship between the pore area and number and the drug release rates, four types of microbullet shells were designed, and we found that the more the pore area and number, the higher the drug release rates. However, the preparation process of microbullets requires higher precision 3D printing machines and professionals, leading to a significant increase in costs. The microbullets were designed specifically for mice. The miniature size of the 3D-printed microbullet shell makes it suitable for oral administration in mice (Fig. 3C).

An important property of the microbullets was enough mechanical strength to ensure smooth and fracture-free penetration into the gastric mucosa. Here, the compressive strength of the microbullet tips was high, reaching >50 N (Fig. 3D&3E), despite the tiny tips. The dental SG resin should be the major reason for providing a high mechanical strength, which has also been evidenced by others [35]. The high mechanical strength of microbullets would likely ensure their successful insertion into the gastric mucosa. Another important property of the microbullets was their stability in the gastrointestinal tract. The swelling rates of the cylinders kept <1.5 % after they were maintained in the simulated gastrointestinal tract environment for 60 h from pH 1.2 and 5.0 to pH 6.8 in turn (Fig. 3F). The microbullets’ minimal volume change may allow them to be freely evacuated from the gut. In the next experiment, we found that the microbullet was completely evacuated from the body. Therefore, the microbullet shell design has several key advantages, including high drug loading and flexible replacement of drugs according to therapeutic needs. The drug release rate can be adjusted by the pores for drug release. In addition, the microbullet shell material is stable, has high mechanical strength, and can resist prolonged compression in the gastrointestinal tract.

### 3.2 Precise and long-term gastric location of the microbullets by magnetic guidance

After the problem of the mechanical strength of microbullets was addressed, the next issue was the microbullet's precise direction. With conventional techniques such as gastric floating and adhesion, the long-term gastric retention of drug delivery systems is a difficult job [36,37], not to mention the localized retention at the pylorus. Additionally, for the therapy of *H. pylori* infection, another huge challenge is the enough antibiotic concentration at the pylorus. The antibiotic resistance of *H. pylori* is gradually increasing because of the rapid emptying of the drugs from the stomach, leading to low concentrations of drugs at the pylorus and the unavoidable abuse of antibiotics. Therefore, the key to the therapy of *H. pylori* infection is the long-term and controlled release of therapeutic drugs at the pylorus to achieve enough bacterial inhibitory concentrations [38].

**Fig. 4:**
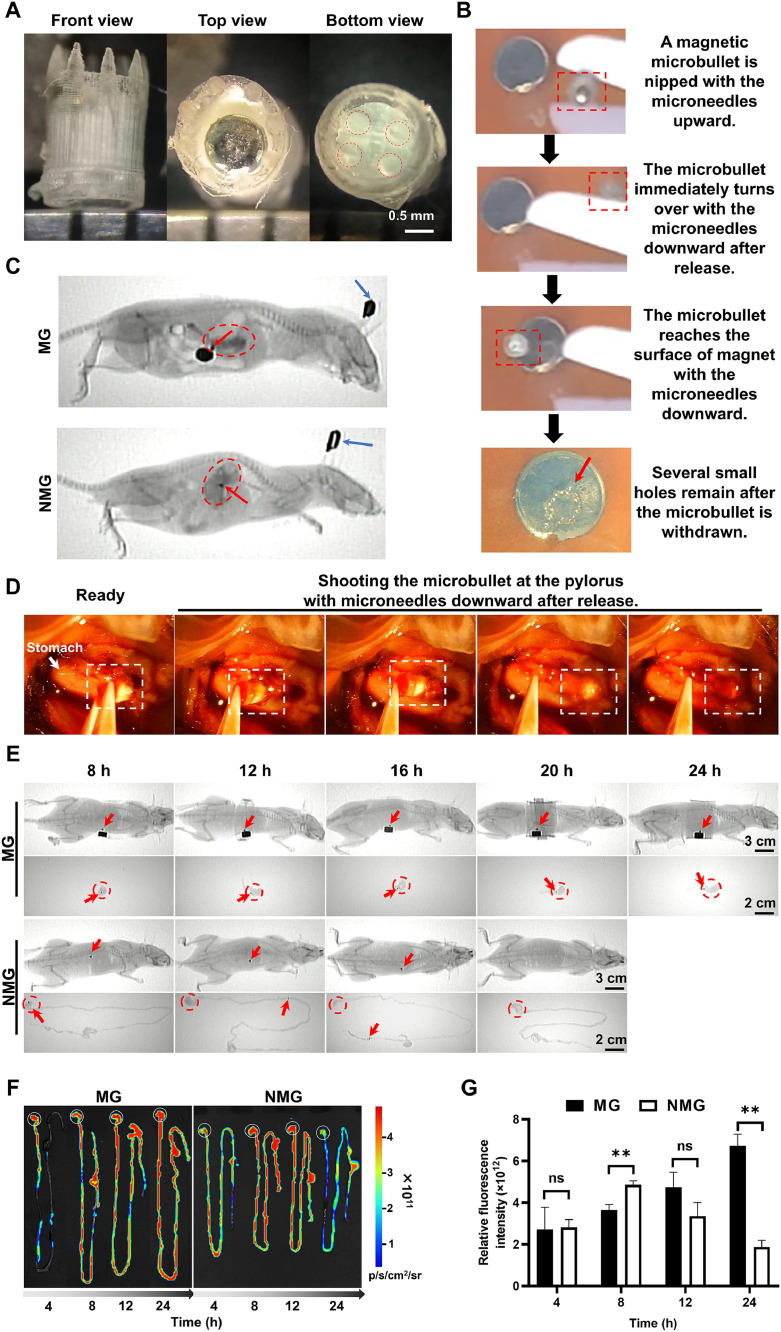
*In vivo* status of oral microbullets. (A) Appearance of the Type 300/4 microbullet. Red circles indicate the pores for drug release. (B) Procedure of shooting a microbullet at an agar brick under magnetic guidance. Red boxes indicate the microbullet. The red arrow indicates the pores pierced by the tip of the microbullet. (C) Images of the microbullet in the stomach. Red circles indicate the stomach. Red arrows indicate the microbullet. Blue arrows indicate ear tags. (D) Procedure of shooting a microbullet at the pylorus under external magnetic guidance. White boxes indicate the microbullet. (E) Retention of the microbullets in the mice in the MG group compared with that in the NMG group. Black patches on the belly of the mice indicate the external magnets. Red circles indicate the stomach. Red arrows indicate the microbullets in the gastrointestinal tract. The images without arrows indicate no microbullet found. (F) Fluorescence images of RMMBs in the gastrointestinal tract. White circles indicate the stomach. (G) Relative fluorescence intensity in the stomach in the MG and NMG groups. *n* = 3. **P < 0.01; ns: no significance.

To make drug delivery systems reach the pylorus and maintain it for a long time, we applied the magnetic guidance method, which was a remote, untethered, and contact-free control of the movement and location of an object via magnetic force. We assembled a polarizing magnet at the top of the microbullet after the drugs had been loaded (Fig. 4A). The microbullet with the tips up quickly turned over and shot into the agar brick under magnetic guidance. The significant pores were left on the brick due to the presence of the microbullet tip microneedles (Fig. 4B, Video 1 in the supplementary materials). In the *in vivo* experiment, if no magnetic guidance (*i.e.*, the NMG group), the microbullet only freely remained in the stomach (Fig. 4C). However, if a permanent magnet was put on the surface at the low position of the stomach (*i.e.*, the MG group), the microbullet was immediately attracted at the site of the external magnet and remained for very long time. Video 2 in the supplementary materials showed that the withdrawn microbullet quickly shot into the pylorus under external magnetic guidance (Fig. 4D). The microbullets in the NMG group remained in the stomach for about 8 h, appeared in the jejunum at 12 h, and the rectum at 16 h, and finally disappeared in the gastrointestinal tract at 20 h (Fig. 4E); therefore, the microbullets in the NMG group only had the GRT of 8–12 h. In contrast, the microbullets in the MG group remained in the stomach for at least 24 h, *i.e.*, the GRT of >24 h. Moreover, the GRT of the microbullets can be effectively prolonged with the aid of external magnetic force. The long-term gastric retention of the microbullets built up a good basis for the continual release and maintenance of the effective local concentration of drugs.

We further evaluated the *in vivo* distribution of drugs with a fluorescent probe, RhB. In the NMG group, the fluorescence was distributed in almost all the gastrointestinal tract and maintained a high intensity in the stomach at 8 h and 12 h (Fig. 4F&4G). However, the fluorescence nearly disappeared in the gastrointestinal tract at 24 h, indicating that no microbullet existed in the tract. However, in the MG group, the fluorescence in all stomachs gradually increased with time due to the continual RhB release. More importantly, the fluorescence maintained a high intensity in the stomach up to 24 h, which should be related to the long-term retention of the microbullets under magnetic guidance. Therefore, the magnetic guiding microbullet may be an optimal formulation for the eradication of *H. pylori* in the stomach.

### 3.3 High *in vivo* anti-*H. pylori* ability of CMMBs

**Fig. 5:**
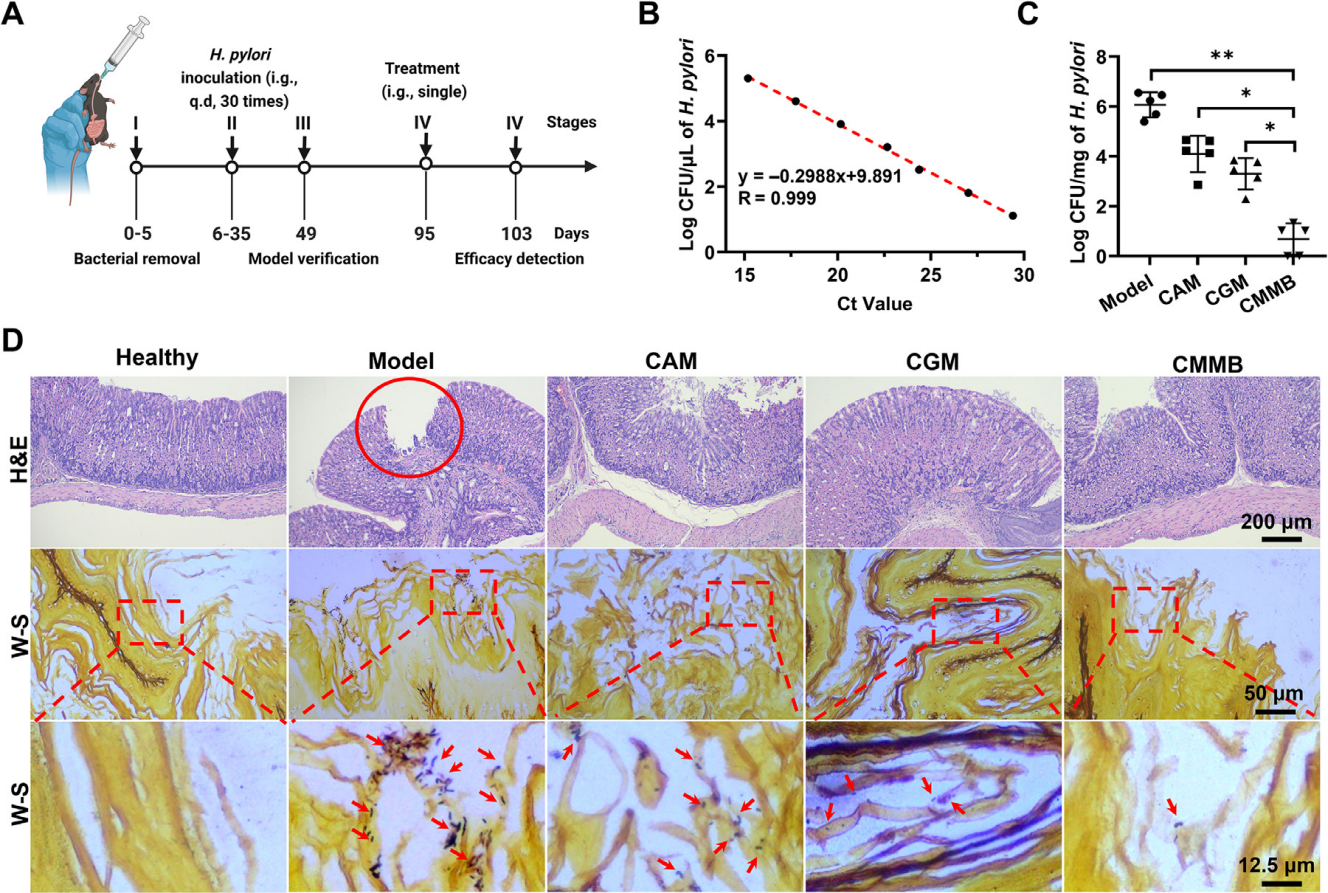
Treatment efficiency of *H. pylori* infection. (A) Schedule of the pharmacodynamics study against *H. pylori* infection. (B) Standard curve of *H. pylori* Ct values. (C) Quantification of *H. pylori* in the stomach tissues on Day 103 (*n* = 5). (D) Images of the H&E and W-S stained gastric tissue sections on Day 103. Red circles indicate gastric mucosal damage. Red arrows indicate the black rod-shaped *H. pylori*. *P < 0.05, **P < 0.01.

We used three methods to evaluate the *H. pylori* eradication efficiency of oral formulations, involving the quantification of *H. pylori* in the stomach, the histopathology of gastric tissues, and the observation of *H. pylori* in the stomach. The quantification of *H. pylori* was realized according to the Ct values (Fig. 5B). After treatment, *H. pylori* in the CAM and CGM groups was 10^4.10^ CFU/mg and 10^3.30^ CFU/mg, respectively, lower than that (10^6.06^ CFU/mg) in the model group (*P* < 0.01). Moreover, *H. pylori in* the CMMB group was only 10^.^^0.^^69^ CFU/mg, much lower than those in the CAM, CGM, and model groups (Fig. 5C, *P* < 0.05). *H. pylori* infection seriously damages the stomach tissues, leading to chronic gastritis or ulcers [39]. The *H. pylori*-infected gastric mucosa tissues in the model group were characterized by the erosion of the lamina propria (Fig. 5D). After CAM treatment, there was no significant erosion though some exfoliated epithelial cells of the gastric mucosa were still present. The treatment of both CGM and CMMB made the stomach tissues almost normalizing with intact gastric mucosal epithelium and lamina propria structures (Fig. 5D). The mucin on the surface of *H. pylori* binds to silver ions, which appear black after being reduced by a chromogenic agent [40]. *H. pylori* was observed in the W-S staining images as a black, short rod, spiral, or curved shape, while the gastric tissue appeared yellow to brown [41]. In the model group, the gastric tissues had numerous black rod-shaped *H. pylori* gathering in the gastric pits. The phenomena were little after the treatment of oral CAM, CGM and CMMBs (Fig. 5D). Moreover, almost no *H. pylori* was observed in the CMMB group, which was consistent with the quantitative results of *H. pylori* in the gastric tissues. Therefore, CMMBs can eradicate the *in vivo H. pylori* only by oral single dosing.

### 3.4 Safety of CMMBs

**Fig. 6:**
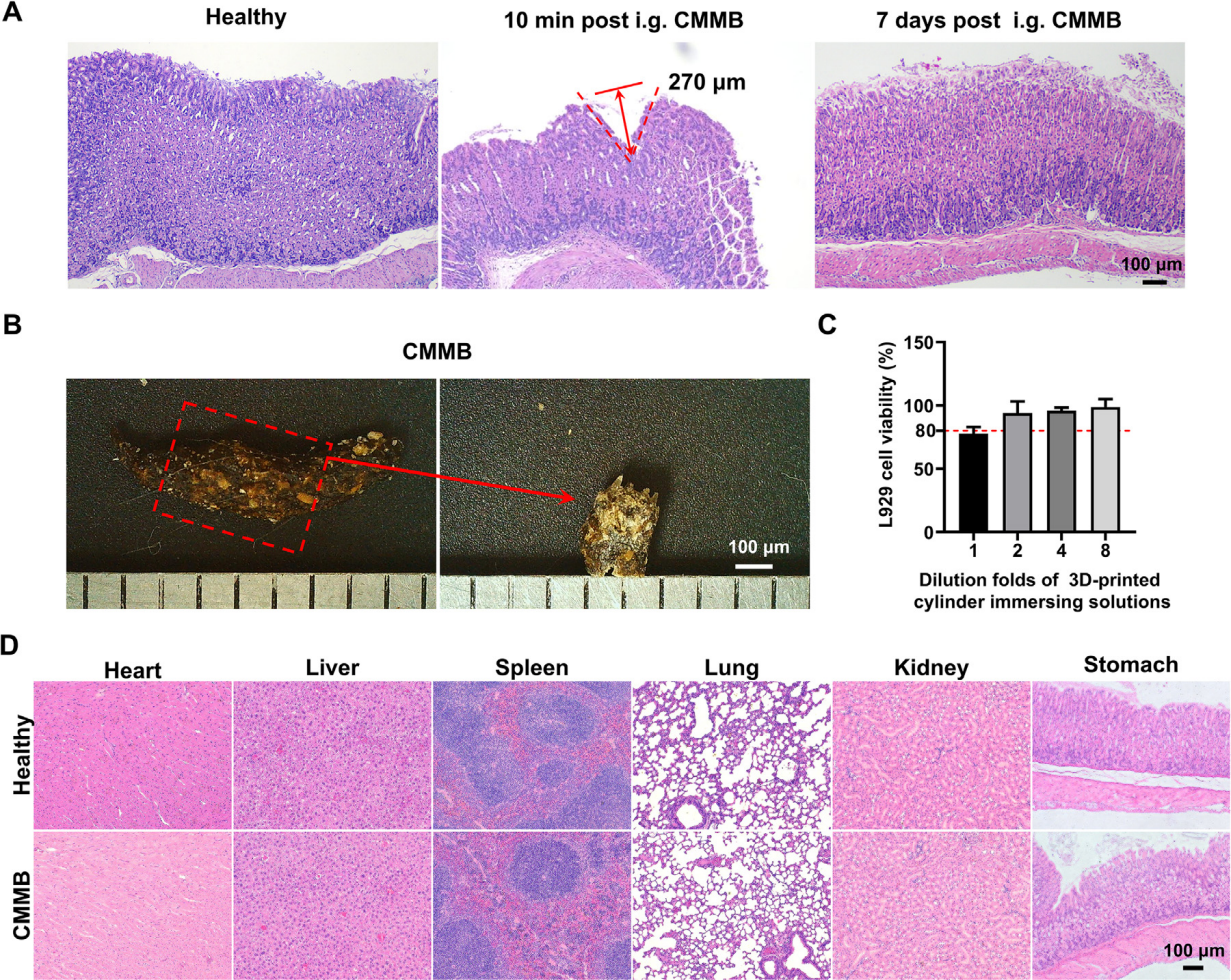
Safety of CMMBs. (A) H&E staining images of gastric tissue sections. (B) Microbullets in the feces. (C) Viability of L929 cells after co-culture with the cylinder-immersing solutions. (D) H&E staining images of the heart, liver, spleen, lungs, kidneys, and stomach tissues following CMMB administration.

The tips of microbullets were inserted into the gastric tissues under magnetic guidance and the depth could be about 270 µm, about 1/3 of the length of the tip (Fig. 6A), which demonstrated the successful design of microbullets. The gastric tissues showed good recovery with the intact tissue layers 7 d post-administration (Fig. 6A). More importantly, the microbullet was completely evacuated with the feces (Fig. 6B). Additionally, the cylinder-immersing solution had little impact on the growth of L929 cells (Fig. 6C). In addition, clinically used combination antibiotics can lessen adverse responses compared to single antibiotics when given the same total dosage [42]. However, the microbullets currently in development have some limitations in realizing multi-drug co-loading due to their small size. Therefore, the microbullet was loaded with only high-dose CAM, *i.e.*, CMMB, and its biocompatibility was evaluated *in vivo*. Following a 36-h retention period of CMMB in the stomach, the major organs (including the heart, liver, spleen, lungs, and kidneys) of the mice showed no significant difference compared to the healthy group, and the gastric tissues showed no accumulation of inflammatory cells (Fig. 6D). Therefore, oral CMMBs are safe.

## 4 Conclusion

*H. pylori* eradication from the stomach is a great challenge in clinics. One-shot successful treatment is attractive because the traditional therapeutic methods are usually very long and the side effects are unavoidable, but it needs enough drug distribution at the pylorus. Two techniques are combined in the treatment of *H. pylori* infections, including 3D printing and magnetic guidance. The 3D-printed hollow microbullet with multiple microneedle tips can realize enough drug loading and deep insertion into the mucosal layer. Magnetic guidance with a magnet outside the body can realize the location of the oral microbullet at the pylorus. Moreover, the local long-term drug release and maintenance of the microbullet make one-shot treatment possible with the complete eradication of *H. pylori* from the stomach. The specific design in this study provides a new opportunity for oral treatment of gastrointestinal diseases with only single-dose administration.

## Conflicts of interest

The authors declare that they have no conflict of interest.

## Appendix Supplementary materials

Image, application 1

Image, video 2

Image, video 3
