# Supplementary material for: Single-dose oral administration of drug-loaded magnetic 3D-printed microbullets for eradication of Helicobacter pylori
Source: Asian J Pharm Sci. 2025 Jan 3;20(2):101013. doi: 10.1016/j.ajps.2024.101013 (PMC11987600; doi:10.1016/j.ajps.2024.101013)
Supplement: Supplementary file 1 [file mmc1.docx]

**Single-dose oral administration of drug-loaded magnetic 3D-printed microbullets for eradication of *Helicobacter pylori***

**1. Methods**

***1.1.*** ***Preparation and characterization of*** ***clarithromycin ground mixtures***

A clarithromycin ground mixture (CGM) was prepared using the grinding method according to a previous report [1]. Briefly, CAM powders were mixed with 2-O-α-D-glucopyranosyl-L-ascorbic acid (AA-2G, Shanghai Aladdin Biochemical Technology Co., Ltd., Shanghai, China) (CAM: AA-2G, 2.2:1.0, w/w), followed by continuous grinding for 30 min. The saturation solubility of CAM and CGM powders in acetate buffer (0.1 mol/L, pH 5.0) was determined with the sulfuric acid color development method as follows: excessive CAM or CGM powders were added to the acetate buffer, followed by shaking at 37 °C and 220 rpm for 24 h. The supernatants were collected after centrifugation at a speed of 14800 rpm for 10 min. Then, sulfuric acid (400 μL) was added to the supernatant (600 μL) to form a yellow solution and the absorbance at 490 nm was detected using a microplate reader (ELX800, BioTek Instruments, Inc., Winooski, USA). CAM concentrations in the solution were calculated according to the CAM linear regression equation. The equation was established as follows: CAM methanol solution (1 mg/mL) was diluted with acetate buffer (0.1 mol/L, pH 5.0) to obtain a series of CAM dilutions. Sulfuric acid (400 μL) was added to the CAM dilution (600 μL) to obtain CAM yellow solutions (5, 10, 15, 20, 25, 30 μg/mL). The CAM standard curve was drawn according to the absorbance at 490 nm of the CAM yellow solutions.

CAM, AA-2G, the physical mixture of CAM/AA-2G (CPM, 2.2:1.0, w/w), and CGM powders were observed under the SEM. The thermal behavior of them was investigated using a differential scanning calorimeter (DSC, TA Q20, TA Instruments Inc., New Castle, USA). The status of CAM in CGM was analyzed using an attenuated total reflection-Fourier transform infrared (ATR-FTIR) spectrometer (Spectrum Two, PerkinElmer, Waltham, USA). The X-ray diffraction (XRD) method was used to investigate the crystalline phase of CAM in CGM with the Cu Kα radiation and the XRD instrument (Rigaku Ultima IV, Rigaku Corp., Tokyo, Japan).

***1.2. Measurement of*** ***CAM release from*** ***microbullets***

A microbullet filled with CAM or CGM powders (eq. to 1 mg of CAM) was placed in 2 mL of acetate buffered solutions (pH 5.0) and shaken at 37 °C and 100 rpm in a thermostatic oscillator. At the predetermined time points (1, 2, 4, 6, 12 and 24 h), an aliquot (600 μL) of dissolution media was withdrawn and supplemented with fresh media of equal volume. The supernatant of each sample was collected after centrifugation at a speed of 14800 rpm for 10 min. CAM concentrations were determined by the sulfuric acid colorimetry as described in Section 1.1.

Drug release curves were fitted to the zero-order, first-order, Higuchi, and Ritger-Peppas kinetics models. The four fitting equations were described as follows:

Zero-order: $\frac{M_{t}}{M_{\infty}}=kt$ (1)

First-order: $\frac{M_{t}}{M_{\infty}}=1-e^{-kt}$ (2)

Higuchi: ${\frac{M_{t}}{M_{\infty}}=kt}^{1/2}$ (3)

Ritger-Peppas: ${\frac{M_{t}}{M_{\infty}}=kt}^{n}$ (4)

where M_t_/M_∞_ was the cumulative amount ratio of released drugs; *t* represents the release time; *k* was the kinetic rate constant; and the release index *n* was a characteristic parameter indicating the release mechanism.

**2. Supporting results and discussion**

***2.1. Characteristics of clarithromycin ground mixtures***

CAM, as a clinically common antibiotic, is always selected as a model drug against *H. pylori* [2-4]. CAM has poor water solubility to impact its application [5]. Hydrophilic modification of CAM may benefit to its release [6]. AA-2G has been proven to improve the solubility of CAM [1]. Here, we mixed CAM and AA-2G to obtain CGM by grinding them thoroughly. To find the unique properties of CGM, we evaluated CGM and the physical mixture of two compounds, *i.e.*, CPM, and the two compounds alone. After grinding, CGM appeared as amorphous powders compared with the crystals of CAM, AA-2G, and CPM (Fig. S1A). Moreover, the FTIR spectra, XRD graphs, and DSC graphs of them indicated that CAM contained amorphous forms (Fig. S1B&C&D). The water solubility of CAM in CGM was highly increased to 2526 µg/mL compared to that of pure CAM with 27 µg/mL (Fig. S1E). The MIC of CGM was 0.5 µg/mL CAM, which was consistent with pure CAM (Fig. S2). The high solubility of CGM provided the opportunity for controlled drug release and high anti-*H. pylori* ability.


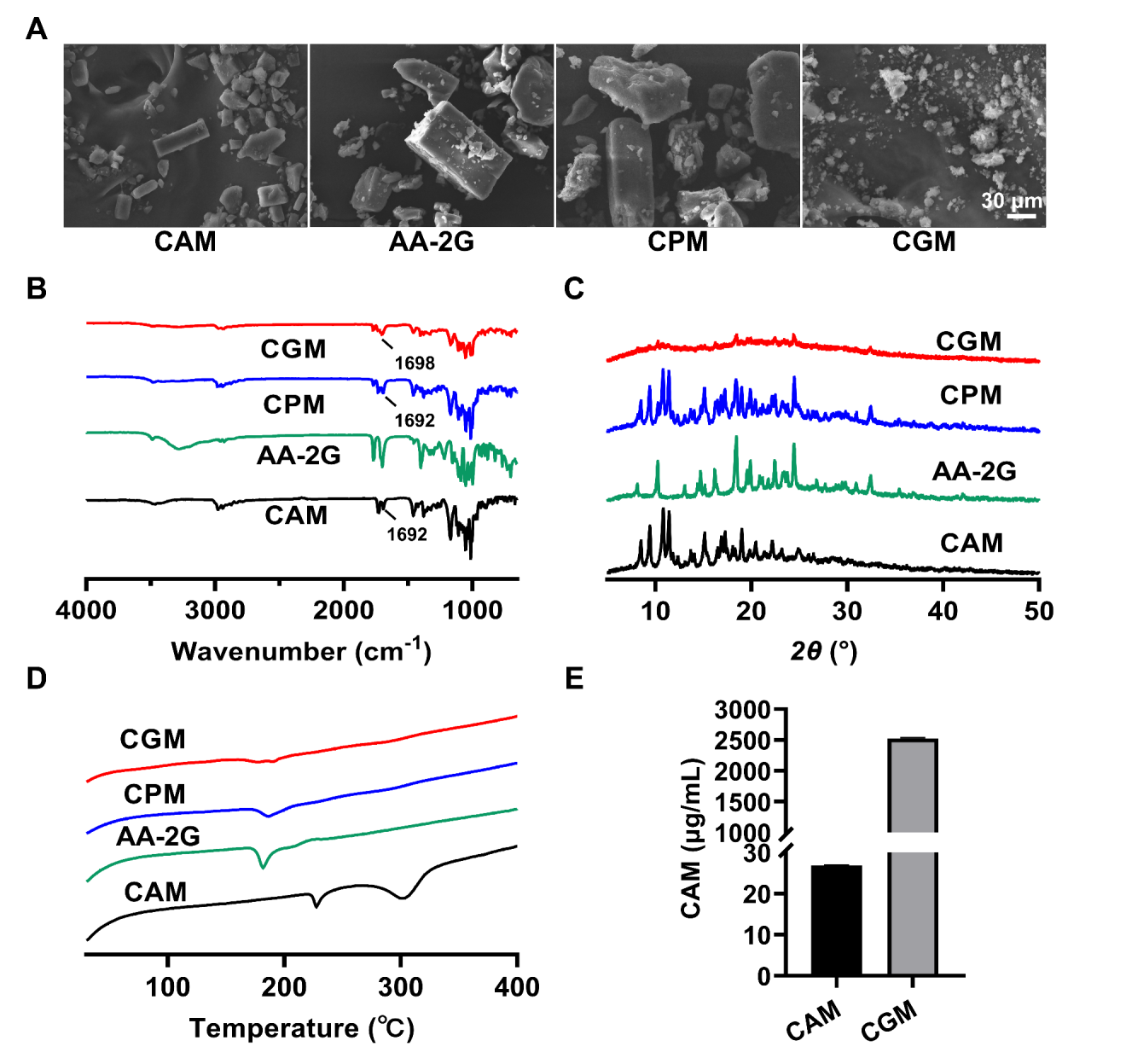


**Fig. S1.** Characteristics of CGM. (A) SEM images. (B) FTIR spectra. (C) XRD graphs. (D) DSC graphs. (E) Saturation water solubility of CAM and CGM at pH 5.0 (*n* = 3).


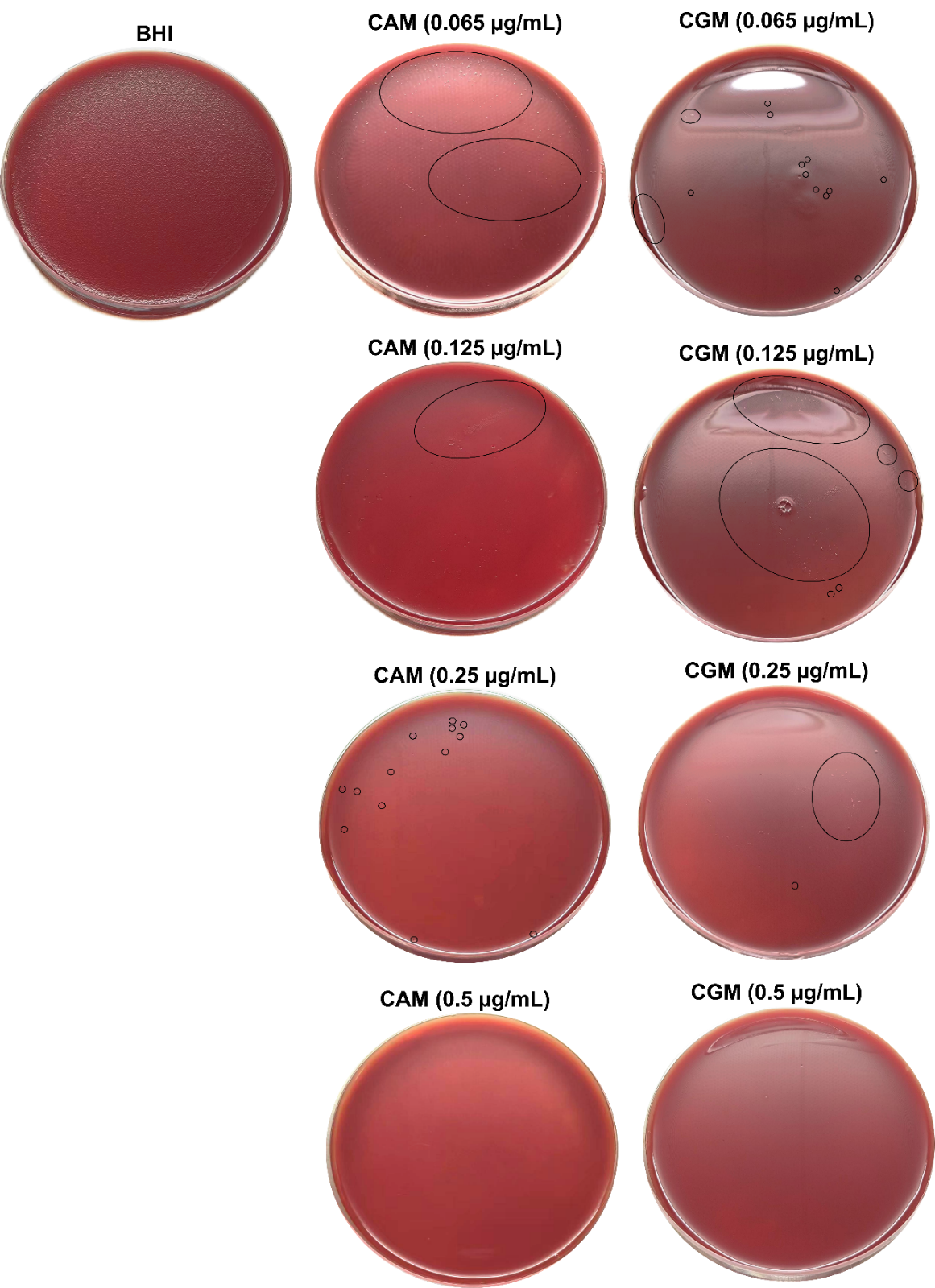


**Fig. S2.** Photos of *H. pylori* colonies on the blood agar plates after treatment with BHI medium, CAM, and CGM solutions, respectively. Numbers in the brackets indicate CAM concentrations. Black circles indicate *H. pylori* colonies. The minimum drug concentration in the sterile growth plate indicates the MIC value of CAM and CGM.

***2.2. Controlled drug release from CMMBs***

The release of water-insoluble drugs is an important factor for their clinical applications [7]. The pores provided the release routes of CAM from the microbullets. The CAM release rates of the pure CAM-loaded and CGM-loaded microbullets were very different. The CAM-loaded microbullets basically showed non-Fickian diffusion release kinetics with the *n* exponents of 0.79, 0.73, 0.74 and 0.78 from the Type 200/2, Type 200/4, Type 300/2 and Type 300/4 microbullet shells, respectively (Fig. S4A). The Type 300/2 and Type 300/4 CGM-loaded microbullets showed first-order release kinetics due to the increase in pore area and solubility, although the Type 200/2 and Type 200/4 CGM-loaded microbullets still showed non-Fickian diffusion release kinetics (Fig. S4B). The release percentages of CAM from the pure CAM-loaded microbullets ranged from 37.11–56.31 % at 24 h for their four types of pore combinations; while the values from the CGM-loaded microbullets were up to 64.25–75.98 % (Fig. S4C). The high concentrations of drugs benefit from eradicating *H. pylori*. The specialized design of the pores structure in the microbullet shell facilitates the sustained release of CAM from microbullets. Moreover, the Type 300/4, *i.e.*, the pore combination with 4 pores and 300 μm in diameter, had the highest release rates among all the microbullets. Therefore, it was chosen as the optimal CMMB for the follow-up studies.


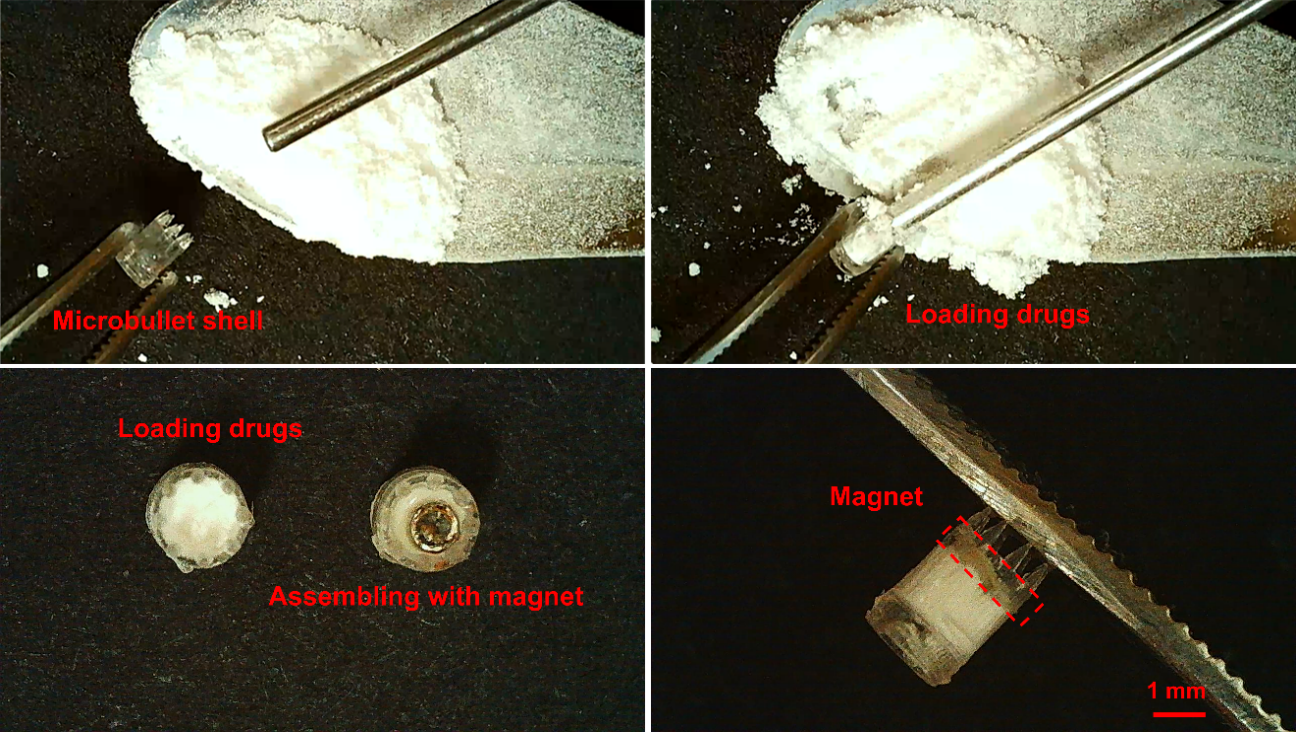


**Fig. S3.** Photos of the assembly process of CMMBs.


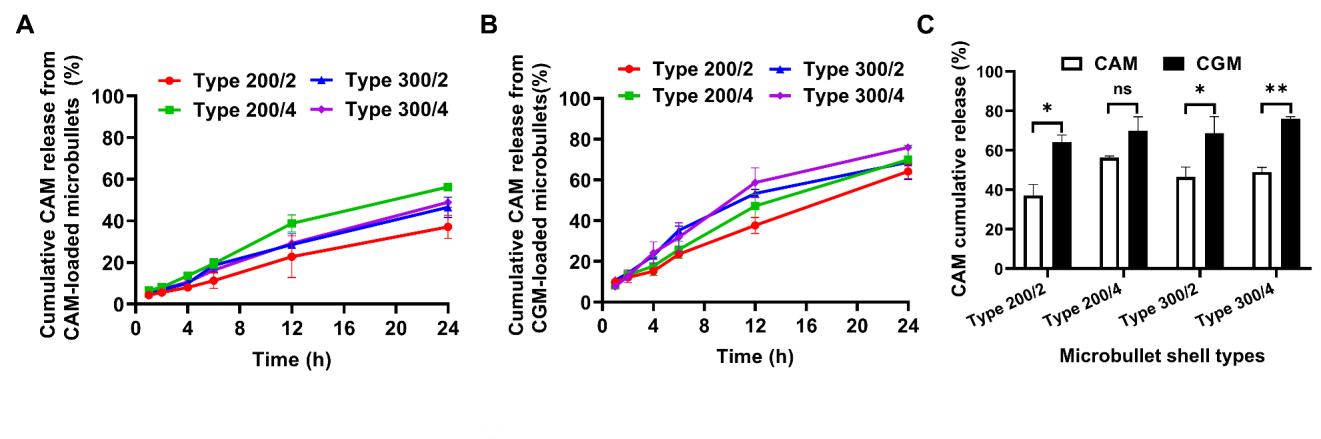


**Fig. S4.** CAM release behavior of CAM-loaded and CGM-loaded microbullets. Cumulative CAM release profiles of the CAM-loaded microbullets (A) and the CGM-loaded microbullets (B). (C) Cumulative CAM release from the CAM-loaded and CGM-loaded microbullets at 24 h. *n* = 3. **P* < 0.05, ***P* < 0.01; ns: no significance.

**References**

[1] Inoue Y., Yoshimura S., Tozuka Y., Moribe K., Kumamoto T., Ishikawa T., et al. Application of ascorbic acid 2-glucoside as a solubilizing agent for clarithromycin: solubilization and nanoparticle formation. Int J Pharm 2007;331(1):38-45.

[2] Chen X., Zou Y., Zhang S., Fang P., Li S., Li P., et al. Multi-functional vesicles improve *Helicobacter pylori* eradication by a comprehensive strategy based on complex pathological microenvironment. Acta Pharm Sin B 2022;12(9):3498-3512.

[3] Hardy D.J. Extent and spectrum of the antimicrobial activity of clarithromycin. Pediatr Infect Dis J 1993;12(12 Suppl 3):S99-S105.

[4] Mosallam F.M., Bendary M.M., Elshimy R., El-Batal A.I. Curcumin clarithromycin nano-form a promising agent to fight *Helicobacter pylori* infections. World J Microbiol Biotechnol 2023;39(12):324.

[5] Nair A.B., Shah J., Al-Dhubiab B.E., Jacob S., Patel S.S., Venugopala K.N., et al. Clarithromycin Solid Lipid Nanoparticles for Topical Ocular Therapy: Optimization, Evaluation and In Vivo Studies. Pharmaceutics 2021;13(4):523.

[6] Liu Y., Chen X., Gao Y., Yu D.G., Liu P. Elaborate design of shell component for manipulating the sustained release behavior from core-shell nanofibres. J Nanobiotechnol 2022;20(1):244.

[7] Li J., Yang Y., Zhao M., Xu H., Ma J., Wang S. Improved oral bioavailability of probucol by dry media-milling. Mat Sci Eng C Mater 2017;78:780-786.
